# AMPA/kainate receptor activation within the prelimbic cortex is necessary for incubated cocaine-craving

**DOI:** 10.3389/fpsyt.2025.1627477

**Published:** 2025-08-14

**Authors:** Laura L. Huerta Sanchez, Mirette G. Tadros, Hoa H. T. Doan, Sylvie V. Vo, Sanil R. Chaudhari, Taylor L. Li, Peter B. James, Audrey Y. Na, Fernando J. Cano, Tod E. Kippin, Karen K. Szumlinski

**Affiliations:** ^1^ Department of Psychological and Brain Sciences, University of California, Santa Barbara, Santa Barbara, CA, United States; ^2^ Department of Molecular, Cellular, and Developmental Biology, University of California, Santa Barbara, Santa Barbara, CA, United States; ^3^ Neuroscience Research Institute, University of California, Santa Barbara, Santa Barbara, CA, United States

**Keywords:** incubation of drug craving, incubation of sucrose craving, prefrontal cortex, AMPA receptors, drug craving

## Abstract

**Introduction:**

The incubation of craving is a behavioral phenomenon in which cue-elicited craving increases during a period of drug abstinence. Incubated cocaine-craving is associated with increased extracellular glutamate within the medial prefrontal cortex (mPFC) and this release, particularly within the prelimbic (PL) subregion, is necessary for incubated cocaine-craving. A potential candidate mediating these incubation-driving effects of glutamate release within the PL are alpha-amino-3-hydroxy-5-methyl-4-isoxazolepropionic acid (AMPA) receptors.

**Methods:**

To investigate the role of mPFC AMPA receptors (AMPARs) in incubated craving, male and female Sprague-Dawley rats were trained to self-administer cocaine for 6 h/day for 10 consecutive days. Either during early or later withdrawal, rats were infused intra-PL with the AMPA/kainate receptor antagonist NBQX (0 or 1 µg/0.5 µl per side), followed by 30-min tests for cue-induced responding. Immunoblotting was also conducted to relate the expression of incubated cocaine- and sucrose-craving to AMPAR subunit expression within mPFC subregions.

**Results:**

Intra-PL NBQX blocked incubated craving expressed in late, but not early, withdrawal. In contrast, an intra-PL NBQX infusion increased cue-induced cocaine-seeking in female rats tested in early withdrawal. No incubation-related changes in AMPAR subunit expression were detected within the PL or IL of rats of either sex and no estrus-associated changes in subunit expression were detected in female rats exhibiting incubated cocaine-craving. In contrast, elevated GluA1 expression was observed within the IL of male rats exhibiting an incubation of sucrose-craving.

**Discussion:**

Together, these findings indicate a necessary role for AMPAR/kainate receptors within the PL in driving incubated cocaine-craving and suggest that AMPAR/kainate receptors located within the IL may be involved also in sucrose-craving selectively in males.

## Introduction

Cocaine use disorder (CUD) is a chronic, relapsing, disorder that leads to devastating behavioral and physical health complications. In 2023, approximately 5 million people in the United States, aged 12 or older, reported using cocaine in the past 12 months. Further, among these individuals, 1.3 million people were diagnosed with a cocaine use disorder in the past year (1). CUD is characterized by a high occurrence of relapse, especially during protracted withdrawal. One factor driving relapse is re-exposure to drug-associated cues. Drug cues induce cravings that can intensify or “incubate” over a period of abstinence, rendering those in recovery more sensitive to the motivational pull of drug-associated cues to drive relapse (2). This so-called “incubation of craving” phenomenon has been demonstrated in both humans and in laboratory animals, the latter of which enables direct investigation of the neurobiology underlying incubated drug-craving (c.f., 3). As summarized in a recent review (3), despite nearly two decades of research focused on the neurobiology of incubated drug-craving, the precise neuroanatomy and molecular mechanisms underpinning incubated drug-craving remain to be elucidated. While a large body of animal research points to the nucleus accumbens (NAc) as an important neural locus in the circuitry underpinning drug craving and it’s incubation during protracted drug abstinence (3, 4), craving induced by exposure to drug-related cues reliably increases prefrontal cortex (PFC) activity in humans with substance use disorders (e.g., 5–7) and increases indices of cellular activity within the medial aspect of the PFC (mPFC) in animal models of incubated drug-craving (e.g., 8–11).

The mPFC is composed of the prelimbic (PL) and infralimbic cortices (IL) and both mPFC subregions project to many reward-related regions, including the nucleus accumbens (NAc) and amygdala (12–15). Further, mPFC subregions receive glutamatergic input from a number of sources, including: strong projections from the basolateral amygdala, the ventral hippocampus and the mediodorsal thalamus, in addition to projections from contralateral mPFC, ipsilateral agranular insular cortex, the claustrum, the midline and paralaminar nuclei of the thalamus and the ventral tegmental area (e.g., 16–20). Supporting a link between incubated cocaine-craving and glutamate hyperactivity within the mPFC, rats expressing incubated cocaine-seeking exhibit a cue-elicited rise in extracellular glutamate within the mPFC that is not apparent in rats tested in early withdrawal (21). Directly implicating this glutamate release as a key driver of incubated cocaine-craving, infusion of a group 2/3 metabotropic glutamate autoreceptor (mGluR) agonist into the PL completely blocks incubated cue-elicited cocaine-seeking, with a measurable, but less robust, effect observed also when the agonist was infused into the IL (22). Our neuropharmacological findings contrast with those obtained under the extinction-reinstatement model of cocaine-seeking in which a dorsal-ventral dichotomy appears to exist with respect to how these two mPFC subregions modulate cocaine-seeking behavior (23–26). However, our observation that glutamate release within both PL and IL subregions contribute to driving incubated cocaine-seeking (22) aligns with optogenetics data implicating the unsilencing of synapses within both PL-NAc and IL-NAc projections in the development of incubated cocaine-craving (27).

Having established that glutamate release within the mPFC is required for the expression of incubated cocaine-craving (22), the question arises as to which postsynaptic glutamate receptors within the mPFC might be mediating the “incubation-driving” effects of cue-elicited glutamate release? Potential receptor candidates may be one or more of the ionotropic glutamate receptors (iGluRs) that rapidly depolarize neurons upon stimulation and mediate “fast” synaptic transmission (c.f., 28). Of the three iGluRs, the expression of kainate receptor (KAR) subunits are altered within several mesocorticolimbic structures following withdrawal from repeated cocaine injections (29), cocaine self-administration (30–32) and in post-mortem tissue from humans with cocaine use disorder (32, 33). Further, null deletion of the gene encoding GluK1 (formerly GluR5) increases sensitivity to the psychomotor-activating and conditioned rewarding properties of cocaine in mice (Gregus et al., 2009), suggesting a suppressive role for KARs in gating behavioral sensitivity to cocaine.

In contrast to the limited information on the role for KARs in cocaine addiction-related behaviors, alpha-amino-3-hydroxy-5-methyl-4-isoxazolepropionic acid receptors (AMPARs) have received considerable attention in the context of incubated drug-seeking, particularly those located within the NAc (c.f., 3, 34). Pharmacological inhibition of AMPARs using the non-selective AMPAR and kainate receptor (KAR) receptor antagonists CNQX or NBQX directly into the NAc reduces cocaine-seeking during protracted withdrawal (e.g., 35–37). However, during protracted withdrawal from daily extended-access to intravenous cocaine, the cell surface expression of the prevalent GluA1 subunit increases within the NAc, along with the synaptic insertion of Ca^2+^-permeable AMPARs (CP-AMPARs) that lack the GluA2 subunit. Supporting CP-AMPAR insertion in incubated cocaine-craving, an intra-NAc infusion of the CP-AMPAR-selective antagonist Naspm blocks cocaine-craving, but only in rats tested in protracted withdrawal when CP-AMPAR expression is high (34, 38). The Naspm effect is sex-independent as it is apparent in both male (34) and female rats (39). Aligning with these data, optogenetics studies implicate the insertion of CP-AMPARs in the maturation of silent synapses within IL-NAc projections during protracted cocaine withdrawal, while the insertion of calcium-impermeable AMPARs contribute to the maturation of silent synapses within PL-NAc projections purported to drive incubated cocaine-seeking behavior (27). While a similar insertion of CP-AMPARs are reported to occur within the PFC of mice injected repeatedly with cocaine (40), to the best of our knowledge, the functional relevance of AMPARs, or any other iGluR, within mPFC subregions for the expression of incubated drug-craving following a period of voluntary self-administration has not been investigated directly.

As a first-pass examination of the role for mPFC iGluRs in incubated cocaine-craving, the current studies examined the effects of an intra-PL infusion of the AMPAR/KAR antagonist NBQX on cue-elicited craving expressed by female and male rats during early versus later withdrawal from a history of long-access (6h/day) intravenous cocaine self-administration. Given the wealth of evidence implicating AMPARs within the NAc in the expression of incubated cocaine-craving (c.f., 3, 34), immunoblotting for GluA1 and GluA2 subunits was also conducted on whole cell lysates of tissue from mPFC subregions as a first pass examination of AMPAR correlates of incubated craving for cocaine versus sucrose. Additionally, we determined how estrous phase might influence AMPAR subunit expression during incubated craving in female rats. To the best of our knowledge, this study is the first to confirm that AMPAR/KAR activation within the PL subregion is required for the expression of incubated cocaine-craving in both female and male rats. Further, we show that the profile of GluA1 and GluA2 expression within whole-cell homogenates from mPFC subregions is distinct between rats expressing incubated cocaine- versus sucrose-seeking of relevance to our neurobiological understanding of these similar behavioral phenomena.

## Materials and methods

### Subjects

Adult male (250-275g) and female (225-250g) Sprague Dawley rats (Charles River Laboratories, Hollister, CA) were housed in a colony room under 12-h reverse light cycle conditions (lights off: 10:00 am). Following arrival, rats were allowed to acclimate to the colony room for 48 h and were given *ad libitum* access to food and water throughout the study. All procedures were approved by the Institutional Animal Care and Use Committee of the University of California, Santa Barbara under protocol number 829 and were consistent with the guidelines of the NIH *Guide for Care and Use of Laboratory Animals*. Note that the rats employed in the immunoblotting study of incubated sucrose-craving were the same rats as those employed in Cano et al. (41). As such, no new animals were required to conduct this experiment.

### Surgery

Under isoflurane anesthesia (4% induction, 1-3% maintenance; Covetrus, Portland, ME), rats were implanted with bilateral guide cannulae (P1 Technologies, Roanoke, VA) aimed above the PL subregion of the mPFC (AP: +3.0; ML: ± 0.75, DV: -2.00 mm from Bregma) and secured to the skull with four stainless steel screws (Specialty Tool, Goleta, CA) and dental acrylic. Rats that were slated to undergo cocaine self-administration were also implanted with a chronic polyurethane catheter (12 cm long; 0.023 inner diameter, 0.038 in outer diameter; Instech Laboratories, Plymouth Meeting, PA) into the right jugular vein and ran subcutaneously over the shoulder to a back incision. The catheter was then secured to a 22-gauge guide cannula (P1 Technologies, Roanoke, VA) in a rat infusion harness (Instech Laboratories, Plymouth Meeting, PA) and capped to protect against infection. Following this procedure, catheters were flushed with 0.1 ml of sterile cefazolin (100 mg/ml) and 0.1 ml of sterile heparin (70 U/ml). Rats slated to be tested for cocaine-craving on WD1 underwent both surgeries on the same day, 7 days prior to the first cocaine self-administration session. Rats slated to be tested on WD30 underwent the IV catheter implantation surgery 5 days prior to cocaine self-administration training procedures and then underwent the intracranial implantation surgery 7 days prior to their test on WD30. Rats were monitored postoperatively for 4–7 days under which rats received subcutaneous Meloxicam (2 mg/kg) once a day for the first 2 postoperative days for pain and daily injections of cefazolin and heparin to maintain catheter patency. To ensure catheter patency prior to cocaine self-administration training, rats were injected IV with 0.1 ml of sodium Brevital (10 mg/ml).

### Cocaine and sucrose self-administration procedures

CP-AMPAR accumulation and increased glutamate transmission within the NAc appear to require long-access cocaine self-administration procedures to manifest (42). As our prior immunoblotting report failed to detect changes in AMPAR subunit expression following short-access cocaine self-administration procedures (8), the rats in the present study were trained to self-administer intravenous cocaine (0.25 mg per 0.1 ml saline infusion; MilliporeSigma, Burlington, MA) over 10 once-daily 6-h sessions under an FR1 schedule of reinforcement with a 20-sec time-point. As detailed in Cano et al. (41), rats in the sucrose-craving study were trained to respond for delivery of a 45 mg banana-flavored sucrose pellet (BioServ, Flemington, NJ) under comparable conditions. In both cases, each lever press on the active lever resulted in a 20-second tone and light stimulus complex (78 dB, 2kHz) signaling reinforcer delivery. Rats were not able to receive additional infusions or pellets during the cue presentation. To provide a baseline for protein expression, a group of cocaine-naive controls were included in the cocaine immunoblotting study (Controls) that only received the 20-second tone-light stimulus when they depressed the active lever (i.e., no primary reinforcer was available). In all cases, depression of the inactive lever produced no programmed consequences. On the first day of IV cocaine self-administration, rats were capped at 100 infusions to prevent overdose. While the rats slated for the immunoblotting studies did not undergo any lever-press training prior to the start of cocaine self-administration procedures, the rats slated for the neuropharmacological study were first trained to lever-press for the sucrose pellets during two 6-h sessions prior to surgery, as conducted in previous microinjection studies (e.g., 11, 43) to engender more reliable subsequent cocaine self-administration behavior.

### Test for cue-elicited cocaine- and sucrose-craving

At early or later withdrawal time-points, (WD1 or WD3 and WD30-31, respectively) rats were placed back into their assigned operant chambers to undergo a test for cue-elicited cocaine- or sucrose-craving. During this test, an active lever press resulted in the presentation of the same 20-second tone and light stimulus complex as experienced during self-administration training, but no cocaine or sucrose delivery. There were no programmed consequences following the depression of the inactive lever. For the rats slated for the immunoblotting studies, this test was 2-h long and tissue was extracted immediately following the end of the test session (see below). For the rats slated for the neuropharmacological study, the test was 30-min long and conducted immediately following the microinjection. Incubated cocaine- and sucrose-craving was defined as a statistically significant increase in active lever presses during the test in later withdrawal (e.g., WD30 or WD31) versus early withdrawal (WD1 or WD3, depending on the study).

### Microinjection

The local infusion of the non-selective AMPAR/KAR antagonist NBQX (44) into the NAc core reduces cocaine-craving when assessed in protracted withdrawal (e.g., 35–37). Thus, we employed a similar approach to examine the role of these receptors within the PL in incubated craving. For this, NBQX (2,3-Dioxo-6-nitro-1,2,3,4-tetrahydrobenzo[*f*]quinoxaline-7-sulfonamide; Tocris, Minneapolis, MN) was dissolved in 1% DMSO and infused at a dose of 1 µg/side, which is comparable to that employed in other studies of drug-induced behavior (45, 46) and 1% DMSO served as the control infusion. On WD1 or WD30, rats were microinjected bilaterally at a rate of 0.5 µl/minute rate for 1 minute with either NBQX or vehicle (total infusion volume/side = 0.5 µl) and microinjectors left in place for an additional 30 sec prior to removal. at a. Immediately following the microinjection, rats underwent the 30-minute cue test described in Sect. 2.4. In order to assess for any potential effects of NBQX on the consolidation of learning, rats were tested again the following day with no further microinjection. Once both tests were completed, rats were sacrificed, brains were extracted, fixed in 4% PFA and sectioned (30 μm thick) for histological verification of microinjector placement using Nissl staining procedures.

### Immunoblotting

Following the 2-h cue-elicited cocaine-seeking test conducted on WD3 or WD30, brains were extracted and the PL and IL subregions of the mPFC were dissected over ice for immunoblotting. As we wanted the present results to be as comparable to prior immunoblotting studies of incubated cocaine-craving, the methods used for whole-cell tissue homogenate preparation, detection and quantification followed similar procedures as those used previously (8, 47). Due to the large number of experimental groups included in the cocaine immunoblotting study, the tissue was processed separately for male and female rats. As fewer groups were tested in the sucrose immunoblotting study (41), tissue from males and females were run concurrently on the same gels. Unfortunately, we had insufficient tissue at the time of study to determine protein expression of KAR subunits from our cocaine- and sucrose-incubated rats, as such, only AMPAR subunit expression was examined herein. In order to quantify AMPAR subunit expression within our samples, anti-rabbit GluA1 (1:500; Millipore; AB1504) and anti-mouse GluA2 (1:1000 dilution; Synaptic Systems; 182 111) primary antibodies were used. Calnexin expression was used to control for protein loading and transfer (anti-rabbit Calnexin primary antibody 1:1,000 dilution; Enzo Life Sciences; ADI-SPA-860). Following primary incubation, membranes were washed with TBST and incubated in either a goat anti-rabbit IRDye 800 CW secondary antibody (1:10,000 dilution; Li-Cor; 925-3221) or a goat anti-mouse IRDye 680RD secondary antibody (1:10,000 dilution; Li-Cor; 925-68070). Membranes were then washed for a second time and imaged in an Odyssey Fc Infrared Imaging System (Li-Cor Biosciences, Lincoln, NE, USA). Protein expression was quantified using Image Studio. Raw values for each band were normalized to their corresponding calnexin signal and then to the average value of the control group (i.e., Control-WD3 for the cocaine study and WD1-males for the sucrose study). The relative expression of GluA2 versus GluA1 subunits was also calculated and expressed as a function of the appropriate control. Blots exhibiting anomalies were excluded from the final statistical analysis of the data.

### Vaginal cytology

Evidence suggests that the magnitude of incubated cocaine-craving varies as a function of the estrus cycle (48–50) and in hippocampus, the synaptic insertion of CP-AMPARs during inhibitory avoidance learning varies as a function of estrous cycle (51). Thus, we monitored each female’s estrous cycle via vaginal swabbing following each cue test. Vaginal samples were collected by gently swabbing the vaginal canal with a cotton-tipped applicator, soaked in sterile saline. Samples were then smeared onto glass microscope slides, sprayed with a fixative, and stained with giemsa staining procedures. The stage of the estrous cycle was determined based on the presence and morphology of cells. Each smear was categorized into one of estrous phases: proestrus, estrus, and diestrus. Proestrus can be recognized by the abundant presence of small nucleated epithelial cells, and estrus by the abundant presence of non-nucleated cornified epithelial cells. Metestrus is identified by the presence of approximately equal amounts of both small and big nucleated epithelial cells, non-nucleated cornified epithelial cells, and neutrophils. Diestrus is characterized by a low cell density and the presence of neutrophils, with occasional nucleated and almost no cornified epithelial cells. Given the relatively low number of female rats in metestrus, the data from the rats in metestrus and diestrus were combined for data analysis as conducted in prior studies (52).

### Statistical analysis

Data was analyzed using analyses of variance (ANOVAs) to examine for behavioral and biochemical outcomes associated with incubation. For the study of the effects of NBQX on incubated cocaine-craving, the average number of active and inactive lever presses emitted during the cue tests were analyzed using a Treatment (WD1-VEH, WD30-VEH, WD30-NBQX) X Sex ANOVA. The data from the study of the effects of NBQX on responding in early withdrawal were analyzed using a Treatment (VEH vs. NBQX) X Sex ANOVA. For the immunoblotting study of incubated cocaine-craving, the data were expressed as a percentage of the average of the two or three Control-WD3 animals on each gel and analyzed using a Group (Control vs. Cocaine) X Withdrawal ANOVA, separately for male and female rats. The behavioral data from this immunoblotting study were also analyzed using Group X Withdrawal ANOVAs, separately for males and females for consistency. As the immunoblotting study of incubated sucrose-craving did not include a sucrose-naive control, the samples from males and females could be immunoblotting concurrently on the same gel. As such, these data were expressed as a percentage of the average of the three Male WD1 rats on each gel and analyzed using a Sex X Withdrawal ANOVA, as conducted previously (41). For the examination of the effect of estrous phase on behavior and protein expression within female cocaine-experienced rats, the data were analyzed using a Phase (estrus, diestrus, proestrus; no rats were found to be in metestrus) X Withdrawal ANOVA. Significant main effects or interactions in all analyses were further investigated with t-tests or tests for simple effects. Outliers were identified and excluded from the analyses using the ± 1 × IQR (interquartile range) rule, however, in instances where too many outliers were identified, we adopted the ± 3 × IQR rule to ensure that only the most extreme outliers were removed. Alpha was set to 0.05 for all analyses with the exception of the analyses of estrous phase influence on behavior and protein expression in which alpha was set to 0.1 as we had *a priori* predictions that: (1) AMPAR subunit expression would vary with estrous cycle phase (51) and (2) incubated cocaine-craving would be highest in female rats in estrus (e.g., 49). IBM SPSS Statistics software (version 27.0 for Macintosh) was used for all statistical tests, and GraphPad Prism software (version 9.3.1 for Macintosh) was used to create all graphs.

## Results

### Intra-PL NBQX lowers incubated cocaine-craving

The results pertaining to the average behavior of the rats over the course of the last 3 days of the cocaine self-administration phase of the study are presented in **Table 1** (Expt. 1). Analyses of the number of active lever-presses [F(5,33)=0.014, p=0.906], inactive lever-presses [F(5,33)=2,457, p=0.127] and reinforcers earned [F(5,33)=0.079, p=0.861] did not indicate any significant group differences at the outset of testing.

**Table 1:** Summary of the data from the self-administration training phases of each experiment summarized in this report.

Active		Reinforcer	Inactive
Exp. 1- NBQX effect on incubated cocaine-craving			
WD1 VEH	230.3+148.09	107.27+24.14	7.47+1.49
WD30 VEH	133.03+45.63	79.5+15.86	8.47+2.16
WD30 NBQX	159.67+69.58	88.28+13.22	22.49+9.64
Exp. 2 - NBQX effect in early cocaine withdrawal			
WD1 VEH	100.26+17.41	77.86+14.58	13.85+5.56
WD1 NBQX	99.66+11.41	89.03+9.89	2.73+1.06
Exp. 3 - lmmunoblotting: Incubated Cocaine-Craving			
Male:			
WD3 Control	26.55+8.72	18.83+1.99	12.87+5.54
WD3 Cocaine	64.06+11.88	52.53+2.42	6.23+9.48
WD30 Control	18.15+5.21	14.92+1.65	10.35+3.90
WD30 Cocaine	81.4+12.47	74.47+2.35	7.48+12.37
Female:			
WD3 Control	28.17+11.07	7.9+1.91	5.47+1.14
WD3 Cocaine	70.4+9.69	64.93+0.73	3.73+9.69
WD30 Control	7.73+1.30	13.85+0.59	2.73+6.96
WD30 Cocaine	70.74+7.67	65.15+11.18	18.15+5.71
Exp.4 - lmmunoblotting: Incubated Sucrose-Cravi ng			
Male:			
WD1	197.91+11.40	170.64+11.54	12.33+2.68
WD30	170.57+10.08	140.51+7.59	15.55+3.02
Female:			
WD1	184.4+12.00	160.22+9.04	9.95+3.31
WD30	176.55+10.43	140.52+9.26	16.64+4.52

The data represent the means ± SEMs. Note that the data from Expt. 4 are derived from Cano et al. (41).

**Figure 1:**
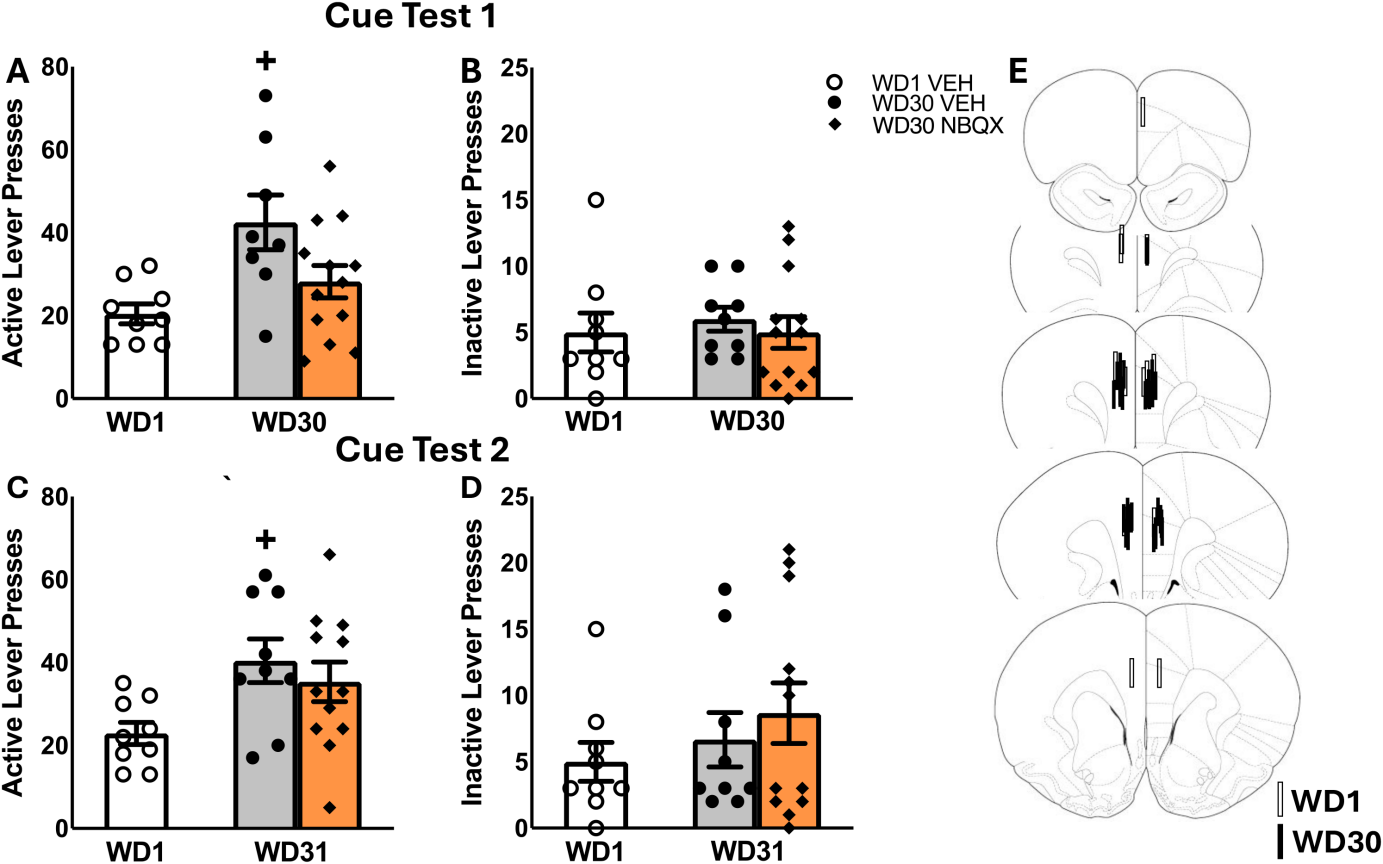
Summary of the effects of an intra-PL infusion of NBQX (1 𝜇g/side) or vehicle (VEH) on active and inactive lever-responding during tests for incubated cocaine-craving conducted either immediately following microinfusion **(A, B)** and during a second test conducted 24 h later **(C, D)**. As no sex differences in responding were detected, the data is collapsed across male and female rats for better visualization of the NBQX effect. The data represent the means ± SEMs of n=9 WD1-VEH, n=8 WD30-VEH and n=12 WD30-NBQX rats. **(E)** Cartoon depicting the placements of the microinjectors within the PL. +p<0.05 vs. WD1 (withdrawal day 1).

A comparison of the number of active (**Figure 1A**) and inactive (**Figure 1B**) lever-presses emitting during a 30-min test for cue-reinforced responding indicated a significant Treatment effect for active lever responding [for active lever: F(5,30)=5.047, p=0.013; for inactive lever: F(5,33)=0.151, p=0.860]. As expected, VEH-infused rats tested on WD30 emitted more active lever-presses than the WD1 controls [t(16)=3.158, p=0.006], indicative of incubated cocaine-craving in the WD30 controls. In contrast, active lever-responding did not differ between NBQX-infused rats tested on WD30 versus VEH-infused rats tested in either early [t(21)=1.264, p=0.220] or later withdrawal [t(19)=.435, p=0.060], indicating that intra-PL NBQX was sufficient to lower cue-reinforced responding on WD30 to block the expression of incubated cocaine-craving.

When tested the next day in the absence of any further pretreatment, we again detected a significant Treatment effect for the number of active lever-presses (**Figures 1C, D**) [for active lever: F(5,29)=4.918, p=0.015; for inactive lever: F(5,30)=.837, p=0.444]. Consistent with our prior studies (e.g., 11, 43, 47), incubated cocaine-craving persisted in VEH-infused rats tested on WD31 [t(16)=3.464, p=0.003]. Cue-reinforced responding of NBQX-pretreated rats did not differ from their VEH-infused counterparts tested in later withdrawal [t(19)=0.814, p=0.484]; however, their responding was now significantly higher than that of VEH-infused rats tested in early withdrawal [t(17)=2.154, p=0.021]. Thus, the inhibitory effect of intra-PL NBQX infusion observed immediately following microinjection (**Figure 1A**) is transient and does not persist into the next day.

Notably, on neither cue test day were sex differences in responding apparent on the active [F(5,29)<2.419, p’s>0.459] or inactive lever [F(5,30)<2.712, p’s>0.111]. Thus, in our hands, the magnitude of incubated cocaine-seeking and its transient blockade by intra-PL NBQX infusion are sex-independent.

### The inhibitory effect of intra-PL NBQX is incubation-selective

To confirm that the reduction in incubated cocaine-craving observed in our initial experiment (**Figure 1A**) was specific to the cocaine-incubated state, we examined the effects of intra-PL NBQX infusion on cue-reinforced responding in rats tested on WD1. The results pertaining to the average behavior of the rats over the course of the last 3 days of the cocaine self-administration phase of the study are presented in **Table 1** (Expt. 2). Analyses of the number of active lever-presses [F(3,20)=0.000, p=0.992], inactive lever-presses [F(3,20)=3.689, p=0.074] and reinforcers earned [F(3,20)=0.462, p=0.507] did not indicate any significant group differences at the outset of testing.

**Figure 2:**
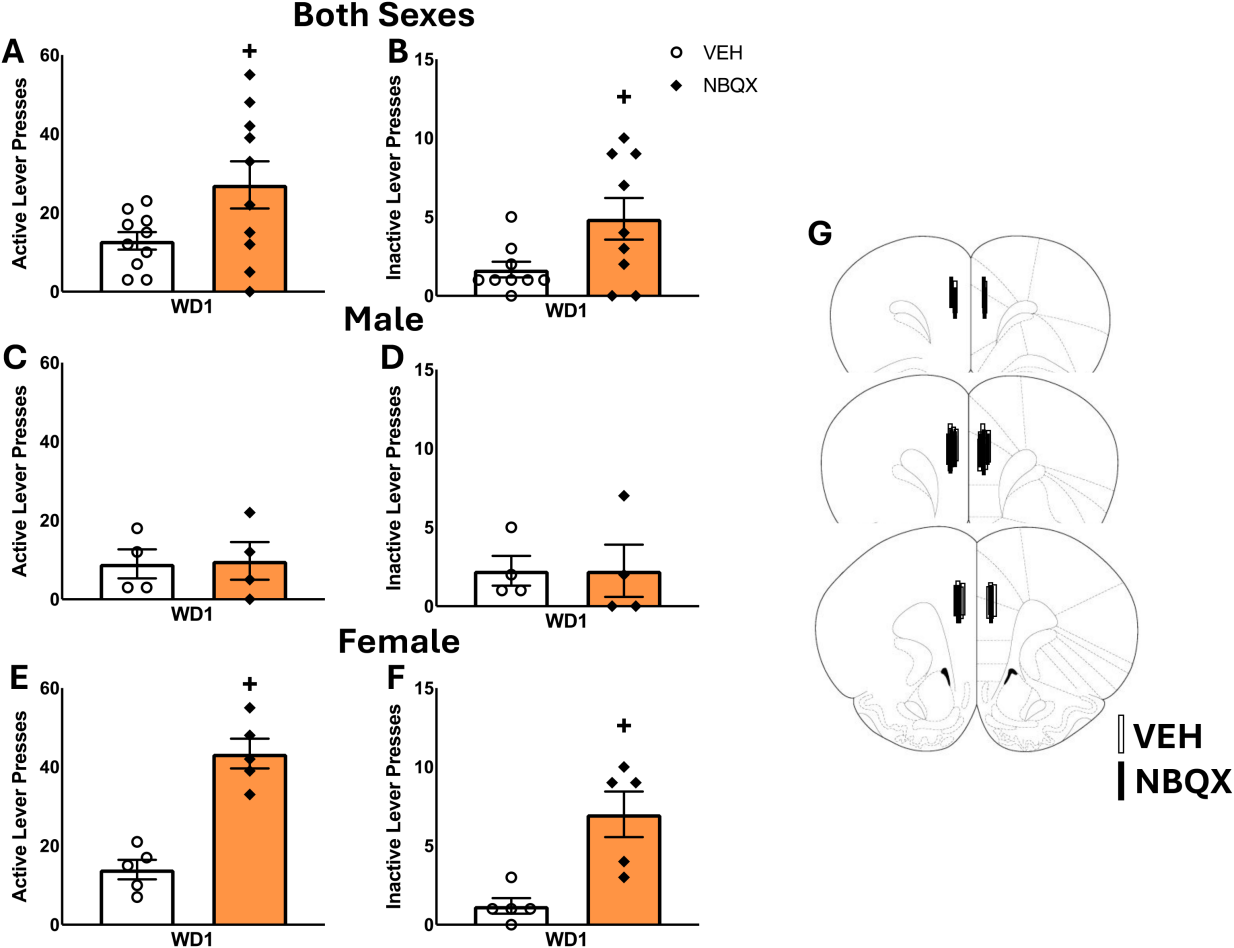
Summary of the effects of an intra-PL infusion of NBQX (1 𝜇g/side) on active and inactive lever-responding during cue tests conducted on withdrawal day 1 (WD1). As a sex difference in the effect of NBQX was detected in this study, the behavior is depicted for both sexes combined **(A, B)**, as well as for males **(C, D)** and females **(E, F)** separately. The data represent the means ± SEMs of n=4 males and n=5 females (total n=9) per group. **(G)** Cartoon depicting the placements of the microinjectors within the PL. *p<0.05 vs. VEH.

Although no sex differences in responding were detected in our study of incubated craving, females emitted more active lever-presses, overall, than males when tested in early withdrawal (**Figures 2A, B**) [Sex effects, for active lever: F(3,20)=23.681, p<0.001; for inactive lever: F(3,18)=2.358, p=0.147]. We also detected significant Treatment effects for both levers [for active lever: F(3,20)= 15.772, p=0.001; for inactive lever: F(3,18)= 5.795, p=.030], as well as significant Treatment X Sex interactions [for active lever: F(3,20)=18.071, p<0.001; F(3,18)=5.795, p=0.030]. Deconstruction of the interaction for active lever-pressing along the Sex factor indicated a robust NBQX-induced increase in cue-reinforced responding by female rats [t(8)=6.50, p<0.001], that was not apparent in males (**Figure 2A**) [t(8)=0.886, p=0.861], with a similar pattern of results observed for inactive lever-pressing (**Figure 2B**) [for females: t(8)=3.792, p=0.005; for males: t(6)=0.000, p=1.000]. Together with our results for incubated cocaine-craving (**Figure 1**), these data indicate that the capacity of NBQX to lower cocaine-craving is selective for the incubated state. Further, the results from this study argue that the effect of intra-PL NBQX infusion on incubated cocaine-craving does not reflect acute motor, motivational or cognitive impairing effects of AMPAR blockade within the PL.

### Immunoblotting for correlates of incubated cocaine-seeking

Given the effects of intra-PL NBQX infusion cue-elicited cocaine-craving and its incubation during protracted withdrawal, we next determined if AMPAR subunit expression within the PL, as well as the more ventral IL subregion, might correlate with behavior. The GluA1 subunit is the most prevalent AMPAR subunit in the brain (e.g., 53). Thus, GluA1 expression was used as a gross index of total AMPAR expression, while the GluA2 subunit and its relative expression were examined to assay potential changes in the number of CP- vs. CI-AMPARs (c.f., 54). The results pertaining to the average behavior of the male and female rats over the course of the last 3 days of the cocaine self-administration phase of the study are presented in **Table 1** (Expt. 3). Analyses of the number of active lever-presses [for females: F(1,27)=0.130, p=0.722; for males: F(1,22)=1.521, p=0.231], inactive lever-presses [for females: F(1,27)=1.918, p=0.178; for males: F(1,22)=0.397 p=0.536] and reinforcers earned [for females:F(1,27)=0.000 p=0.985; for males: F(1,22)=2.729 p=0.113] did not indicate any significant group differences at the outset of testing.

### Cocaine-seeking behavior

**Figure 3:**
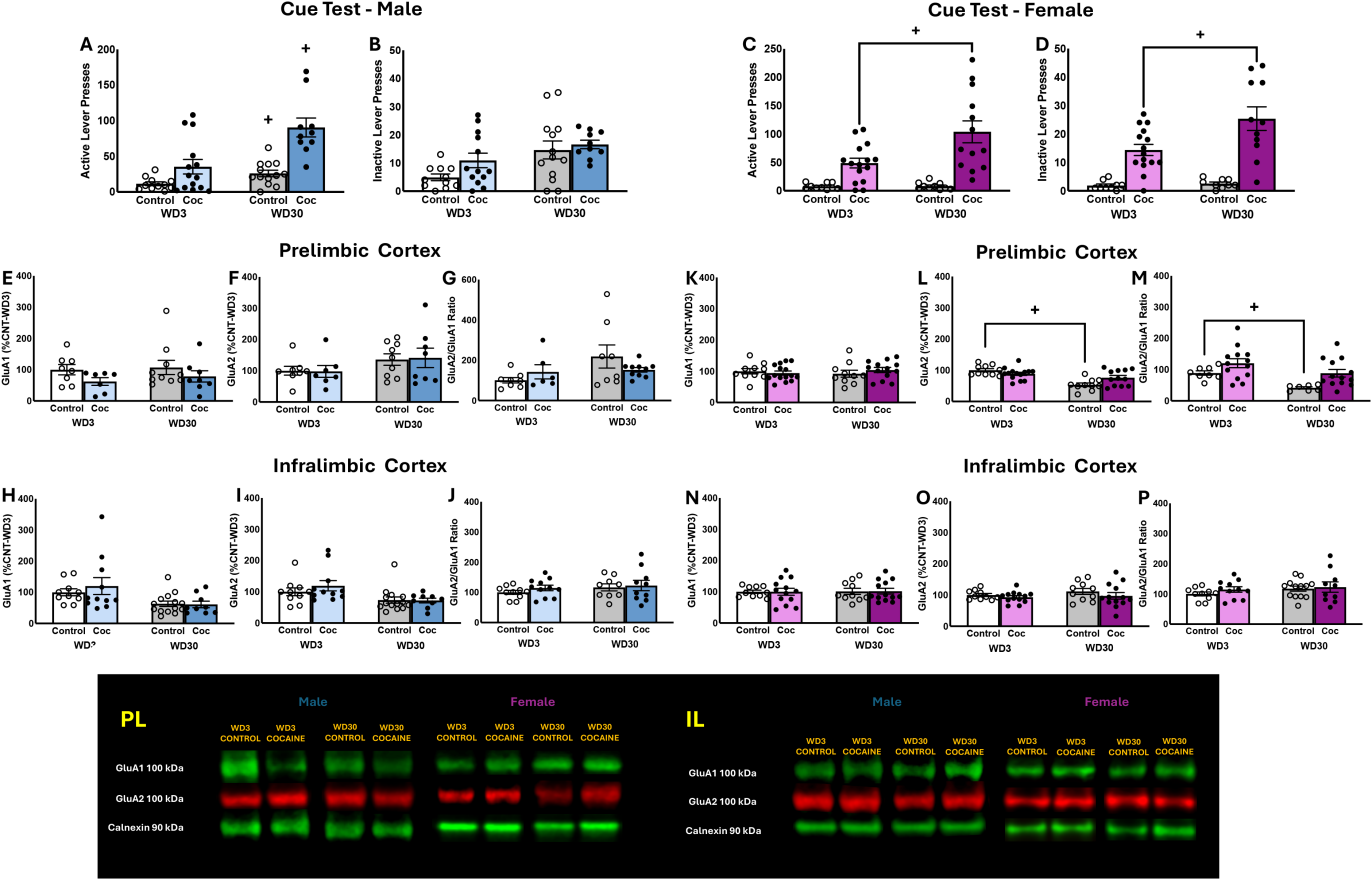
Summary of the behavior exhibited by male **(A, B)** and female **(C, D)** rats during the test for incubated cocaine-craving. Summary of the immunoblotting results for the PL of males **(E–G)** and females **(H–J)**, as well as the immunoblotting results for the IL of males **(K–M)** and females **(N–P)**. The behavioral data represent the means ± SEMs of n=11 WD3-Control, n=13 WD3-COC, n=13 WD30-Control and n=10 WD30-COC males, as well as n=10 WD3-Control, n=15 WD3-COC, n=10 WD30-Control and n=13 WD30-COC females. The immunoblotting data represent the means ± SEMs of n=8–10 WD3-Control, n=6–11 WD3-COC, n=8–14 WD30-Control and n=8 WD30-COC males, as well as n=10 WD3-Control, n=13–15 WD3-COC, n=10 WD30-Control and n=12–13 WD30-COC females. Representative immunoblots are also provided. +p<0.05 vs. WD1.

Significant Group X Withdrawal interactions were detected for the number of active lever-presses emitted during the 2-h cue test by both male (**Figure 3A**) and female rats (**Figure 3C**) [for males: F(1,46)=4.126, p=0.049; for females: F(1,48)=5.169, p=0.028]. In the cases of both sexes, these interactions reflected a time-dependent increase in cue-reinforced responding in the cocaine-experienced rats [for males: t(22)=3.355 p=0.0029; for females: t(26)=2.739, p=0.0110]. Cocaine-naive male controls also emitted more active lever-presses in late versus early withdrawal, but this effect was not detected in female controls [for males: t(22)=2.614,p=0.012; for females: t(18)=0.1546, p=0.879]. While no interaction was detected for inactive lever-responding by male rats (**Figure 3B**) [F(1,47)=.031, p=0.862], the interaction term was significant for females (**Figure 3D**) [F(1,48)=6.086, p=0.018] and reflected a time-dependent increase in inactive lever-responding selectively in the cocaine-experienced animals [for Controls: t(16)=0.8823, p=0.3907; for Cocaine: t(24)=2.602, p=0.016].

### AMPAR subunits within mPFC subregions

A comparison of the total protein expression of GluA1 and GluA2 subunits within the PL of male rats failed to detect any differences for either subunit or for the ratio of GluA2/GluA1 (**Figures 3E–G**) [for GluA1: F(3,45)<1.562, p’s>0.0218; for GluA2: F(3,45)<2.844, p’s>0.056; for GluA2/GluA1 Ratio: F(3,32)<3.646, p’s>0.066]. A comparable analysis of AMPAR expression within the PL of female rats also failed to detect group differences in GluA1 (**Figure 3K**) [F(3,44)<1.079, p’s>0.304]. In contrast, a significant Group effect [F(3,46)=24.320, p<0.001] and Group X Withdrawal interaction [F(3,46)=.8.235, p=0.006] were detected for PL GluA2 expression in female rats (**Figure 3L**). However, deconstruction of the interaction along with Group factor indicated that this interaction reflected a time-dependent reduction in GluA2 expression in the cocaine-naive controls, with no change detected in cocaine-experienced females [for Control: t(22)=2.616, p=.016; for Cocaine: t(24)=1.428, p=0.166]. A Group effect [F(3,39)=7.095, p=0.011] and a Withdrawal effect [F(3,39)=6.673, p=0.013] were detected for the ratio of GluA2/GluA1 within the PL in female rats, that reflected higher relative expression of GluA2 within the PL of cocaine-experienced versus -naive females at the WD30 time-point (**Figure 3M**).

In contrast to the PL, a time-dependent decrease in both GluA1 and GluA2 expression was observed within the IL of male rats (**Figures 3H, I**) [for GluA1: F(3,44)=8.628, p=0.005; for GluA2: F(3,44)=8.725, p=0.005]. Although it appeared that the Withdrawal effects for GluA1 and GluA2 expression were driven, respectively, by the cocaine-naive controls and the cocaine-experienced males, we detected no significant Group effects or Group X Withdrawal interactions for either subunit [for GluA1: F(3,44)<0.462, p’s>0.500; for GluA2: F(3,44)<0.696, p’s>0.409]. These findings align with a failure to detect changes in the ratio of GluA2/GluA1 within the IL of male rats [for GluA2/GluA1 ratio: F(3,44)<1.421, p’s>0.240] (**Figure 3J**). No changes in GluA1 expression, GluA2 expression, and the GluA2/GluA1 ratio were observed within the IL of female rats (**Figures 3N–P**) [for GluA1: F(3,47)<0.019, p’s>0.893; for GluA2: F(3,46)<1.694, p’s>0.199; for GluA2/GluA1 ratio: F(3,45)<3.351, p’s>0.074]. Taken together, these data argue that the expression of incubated cocaine-craving is not associated with changes in the total protein expression of GluA1 or GluA2 subunit expression within either mPFC subregion.

### The influence of estrous phase on incubated cocaine-craving and AMPAR expression

**Figure 4:**
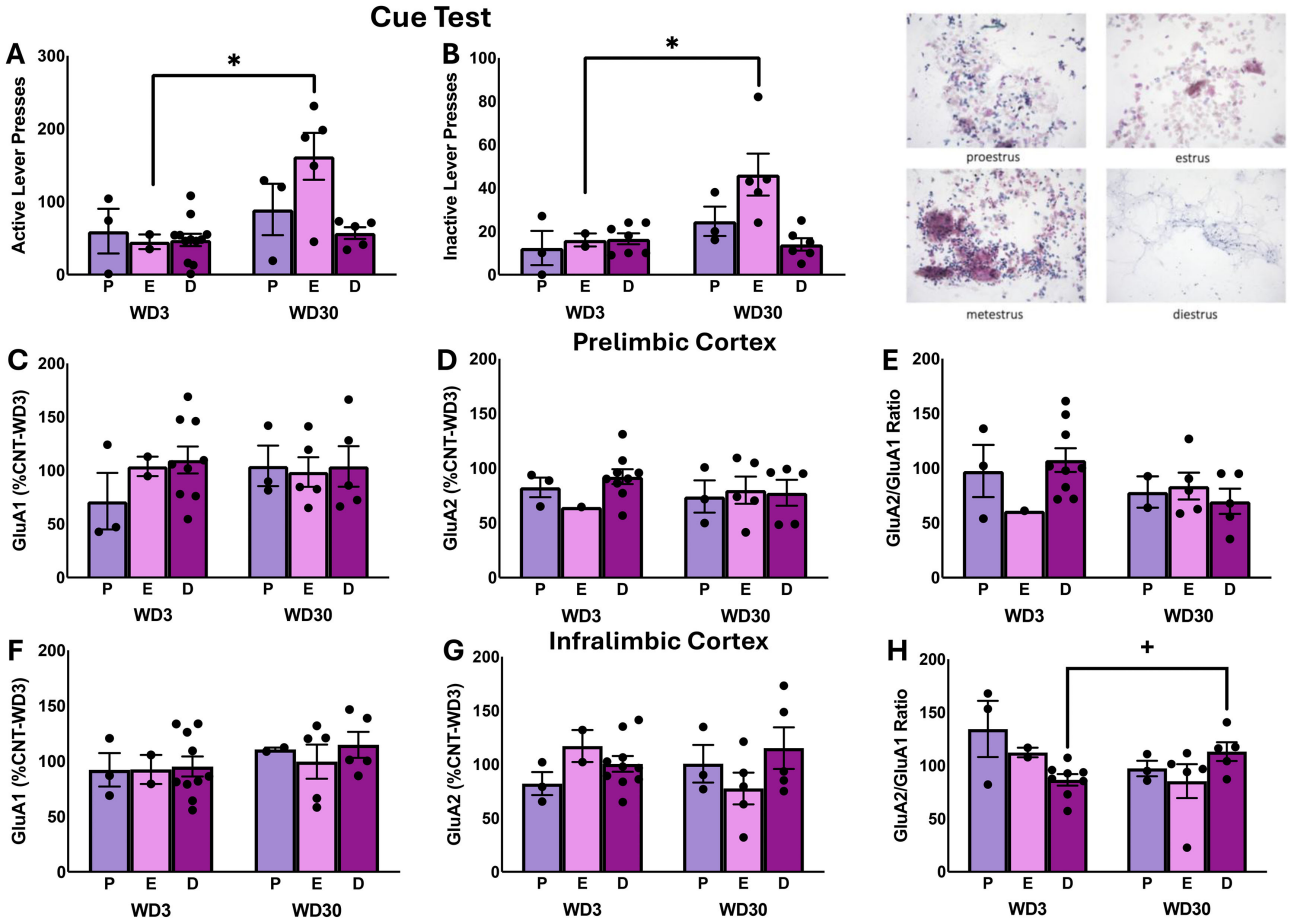
Table: Comparison of responding of female rats in proestrus (P), diestrus (D) and estrus (E) during the test for incubated cocaine seeking **(A, B)**, as well as protein expression within the PL **(C–E)** and the IL **(F–H)**. The data represent the means ± SEMs of n=3 P females per withdrawal time-point, n=2 E-WD3, n=5 E-WD30, n=9 D-WD3 and n=5 D-WD30. * p<0.05 WD3 vs. WD30 (incubation effect). Representative images of the distinctions in vaginal cell cytology across the different phases of the estrous cycle.

Prior studies indicated that the magnitude of incubated cocaine-craving expressed by female rats varies as a function of the estrous cycle (48–50). Although we did not detect any overt sex differences in the magnitude of incubated craving in the present study (**Figure 1**), we wanted to see if behavior and subunit expression might fluctuate with estrous phase in cocaine-experienced females as reported previously in the literature (α=0.1 for these analyses). Indeed, the number of active lever-presses varies with estrous cycle phase in cocaine-experienced females (**Figure 4A**) [Stage effect: F(5,28)=2.638, p=0.094; Withdrawal effect: F(5,28)=7.210, p=0.014; interaction: F(5,28)=2.918, p=0.075]. The Stage effect reflected higher responding in estrus females than those in diestrus (p=0.001) or proestrus (p=0.042), while deconstruction of the interaction along the Stage factor indicated that only estrous females exhibited higher responding on WD30 vs. WD1 [α=0.1; estrous t(5)=2.172, p=0.082; diestrous t(13)=1.058, p=0.309; proestrous t(4)=-0.636, p=.560). Inactive lever-pressing behavior also varied with estrous phase (**Figure 4B**) [Stage effect: F(5,27)=3.532, p=0.048; Withdrawal effect: F(5,27)=6.681, p=0.017; interaction: F(5,27)=3.007, p=.071]. This Stage effect also reflected differential responding by estrus females versus diestrus (p<0.001) and proestrus females (p=0.011) and deconstruction of the interaction for inactive lever-pressing that only estrus females exhibited higher responding on WD30 vs. WD1 (α=0.1; estrous t(5)=2.993, p=0.033; diestrous t(12)=0.514, p=0.616; proestrous t(4)=1.187, p=.301.

The estrous cycle-related changes in the behavior of cocaine-experienced females were not accompanied by any overt estrous cycle-related effects on GluA1 expression, GluA2 expression, or GluA2/GluA1 ratio within the PL (**Figures 4C–E**) [for GluA1: F(5,27)<1.394, p’s>0.250; for GluA2: F(5,26)<0.554, p’s>0.583; for GluA2/GluA1 ratio: F(5,25)<1.284, p’s>0.300] or the IL of cocaine-experienced female rats (**Figures 4F, G**) [for GluA1: F(2,27)<0.617, p’s>0.548; for GluA2: F(5,26)<1.902, p’s>0.172]. A significant Stage X Withdrawal interaction [F(5,25)=4.097, p=0.032] was detected for the ratio of GluA2/GluA1 subunit expression within the IL of cocaine-experienced female rats and deconstruction of this interaction by Stage demonstrated a time-dependent increase in the ratio of GluA2/GluA1 subunit expression in cocaine-experienced rats only in the diestrus phase (**Figure 4H**) [diestrus: t(11)=2.735, p=0.019; estrus: t(5)=1.008, p=0.409; proestrus: t(4)=1.354, p=0.247].

### Immunoblotting for correlates of incubated sucrose-seeking

**Figure 5:**
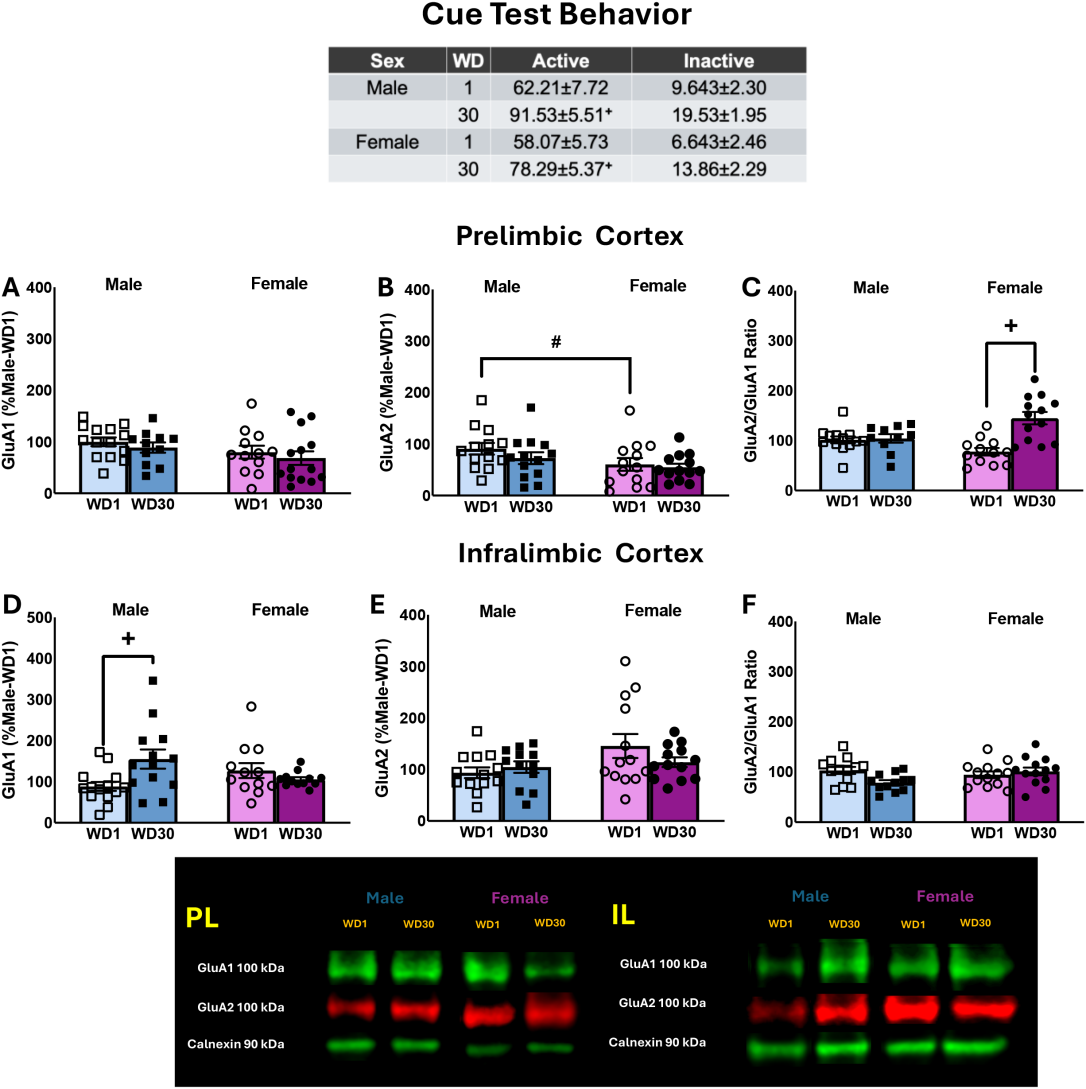
Table: Summary of the behavior exhibited by male and female rats during the test for incubated sucrose-craving, as well as the immunoblotting results for the PL **(A–C)** and IL **(D–F)**. The data represent the means ± SEMs of n=13–14 WD1-males, n=11–13 WD30-males, n=12–13 WD1-females and n=13–14 WD30-females. Representative immunoblots are also provided. +p<0.05 vs. WD1; #p<0.05 male vs. female.

The results pertaining to the average behavior of the rats over the course of the last 3 days of the sucrose reinforcement phase of the study are presented in **Table 1** (Expt. 4) and the results of the statistical analyses described in Cano et al. (41). The table in **Figure 5** summarizes the behavioral results from the tests for incubated sucrose-craving, in which rats of both sexes exhibited comparable incubated sucrose-craving during protracted withdrawal, as well as increased responding on the inactive lever (41). As all rats in this study were sucrose-experienced, the data are expressed relative to the male rats tested for sucrose-seeking in early withdrawal. Overall, females tended to exhibit higher GluA1 expression within the PL (**Figure 5A**) [Sex effect: F(3,47)=3.189, p=0.081; Withdrawal effect and interaction: F(3,47)<1.216, p’s>0.275] and the sex difference in GluA2 expression was statistically significant (**Figure 5B**) [Sex effect: F(3,48)=5.031, p=0.030; Withdrawal effect and interaction: F(3,47)<0.994, p’s>0.323]. A significant Withdrawal effect [F(3,51)=12.124, p=0.001] and Sex x Withdrawal interaction [F(3,51)=15.939, p<0.001] were detected for the ratio of GluA2/GluA1 subunit expression within the PL (**Figure 5C**). This interaction reflected a time-dependent increase in the ratio of GluA2/GluA1 in female, but not male, sucrose-seeking rats [for females: t(23)=4.559, p<0.001; for males: t(24)=0.437, p=0.666].

When subunit expression was compared within the IL, we detected a significant Sex X Withdrawal interaction for GluA1 (**Figure 5D**) [F(3,56)=4.496, p=0.039] that reflected a time-dependent increase in GluA1 in male, but not female, sucrose-seeking rats [for males: t(26)=-2.030, p=0.053; for females: t(26)=0.930, p=0.361]. No group differences in GluA2 expression or the ratio of GluA2/GluA1 expression were observed within the IL (**Figures 5E, F**) [for GluA2: F(3,56)<2.018, p’s>0.161; for GluA2/GluA1 ratio: F(3,56)<1.646, p’s>0.205]. Thus, the expression of incubated sucrose-craving is associated with increased GluA1 expression within the IL, at least in male rats.

## Discussion

Ionotropic glutamate receptors, in particular AMPARs, are considered critical biomolecular mediators of synaptic plasticity, including that associated with withdrawal from repeated cocaine exposure (34, 54, 55). Despite this, and considerable evidence from human imaging studies implicating mPFC hyper-activation in drug cue-reactivity that can drive craving (e.g., 5–7), few studies have directly examined the role for AMPAR within the mPFC in the intensification of drug cue-reactivity that occurs during protracted cocaine withdrawal (40) and none have directly examined the role for KARs (56). Herein, we show that an intra-PL infusion of the AMPAR/KAR antagonist NBQX is sufficient to block the expression of incubated cocaine-craving expressed by both female and male rats tested in later withdrawal. In contrast, intra-PL NBQX infusion produces the opposite effect in female rats tested in early withdrawal and increases cue-reinforced responding. Despite these neuropharmacological results implicating AMPAR/KAR activation within the PL as important for modulating cue-elicited cocaine-craving, we failed to detect any cocaine- or cocaine incubation-related changes in GluA1 or GluA2 subunit expression within either the PL or IL subregion under conventional immunoblotting procedures using whole-cell lysates. In contrast, the expression of incubated sucrose-craving was associated with an increase in GluA1 expression within the IL of male rats only. Below, we discuss these findings within the context of the limited literature focused on the role for glutamate transmission within the mPFC in cocaine- and sucrose-craving, as well as their incubation during protracted reinforcer abstinence.

### Inhibition of PL AMPAR/KARs blocks incubated cocaine-craving

Incubated cocaine-craving is associated with a cue-elicited increase in extracellular glutamate within the mPFC (21) and this glutamate release, particularly within the PL subregion, is necessary for incubated cocaine-craving (22). The current studies demonstrate that AMPAR/KAR inhibition within the PL by NBQX blocks incubated cocaine-craving, without impacting responding on the non-reinforced, inactive, lever (**Figure 1**). Our collective findings argue that cocaine cue-elicited glutamate release during late withdrawal is activating AMPAR and/or KARs within the PL to drive incubated cocaine-craving. Thus, as reported for the NAc (34, 38, 57), AMPAR/KAR stimulation within the mPFC also plays a necessary role in the expression of cocaine-craving following a period of cocaine-abstinence. While providing novel evidence that the activation of iGluRs within the PL drives incubated cocaine-craving, the precise identity of the receptors involved cannot be discerned from the results of this study as NBQX inhibits both AMPAR and KAR function (e.g., 44). Thus, an important next step in future work is to employ selective antagonists of AMPARs (e.g., CP-465,022 or perampanel; 58, 59) or KARs (e.g., UBP296 or ACET; 60, 61) to parse their relative contributions to incubated cocaine-craving.

Herein, the effect of NBQX within the PL on cocaine-seeking was selective for the incubated state as NBQX infusion did not lower cue-conditioned responding in early withdrawal in rats of either sex (**Figure 2**). Although NBQX infusion did not affect responding on the non-reinforced (inactive) lever during the test for incubated craving on WD30 (**Figure 1**), NBQX infusion *increased* responding by female rats on both the cocaine cue-reinforced and non-reinforced (inactive) lever when tested on WD1 (**Figures 2C–F**). The precise reason why an intra-PL infusion of NBQX nondiscriminately increased lever-pressing behavior at this time-point and only in females is unclear at present as we are unaware of any published report examining the effects of intra-PFC NBQX infusion on operant behavior, although an intra-PFC infusion of the CP-AMPAR antagonist Naspm is reported not to alter response inhibition during a stop-signal task (42). The majority of rodent studies indicate that systemic or intracranial pretreatment with NBQX or more selective AMPAR antagonists does not alter spontaneous or stimulant-induced locomotor activity in rodents (e.g., 62–65; but see 66, 67) or influence inactive lever-pressing during early or later withdrawal in studies of incubated drug-craving (38, 68, 69). This being said, selective inhibition of TARP γ-8-bound AMPARs upon systemic pretreatment with JNJ-55511118 was reported to increase inactive lever-pressing behavior in a study of alcohol reinforcement and this effect was apparent in both male and female alcohol-reinforced mice. Curiously, systemic JNJ-55511118 did not elevate inactive lever-responding in a parallel study of sucrose reinforcement nor were any effects observed on general locomotor activity, leading to the conclusion that inhibition of TARP γ-8-bound AMPARs impairs operant response-reinforcer contingency in an alcohol-dependent manner (70). Whether or not the NBQX-induced increase in both active and inactive lever-responding on WD1 reflects a disruption of response-cue reinforcer contingency in early cocaine withdrawal related to inhibition of TARP-bound AMPARs in female rats requires direct examination using selective TARP-bound AMPAR antagonists and should be considered given the purported role for TARPs in the trafficking of AMPARs within the NAc in incubated cocaine-craving (71).

Alternatively, or additionally, the NBQX-induced increase in lever-pressing by female rats on WD1 may reflect factors related to KAR inhibition within the PL, based on evidence that constitutive knock-out of the gene encoding GluK1 increases sensitivity to the psychomotor-activating and conditioned rewarding properties of cocaine in mice (Gregus et al., 2009). As this study employed a single, relatively high, dose of NBQX (45), it remains to be determined whether the female-selective effect of NBQX on WD1 reflects sex differences in the affinity of AMPARs or KARs for NBQX or in the baseline activity or expression of these receptors within the PL. While not yet assessed within mPFC, KAR density is lower in the CA1 region of the hippocampus of estrus females, compared to males (72), raising the possibility that the sex difference in lever-pressing behavior on WD1 might reflect lower KAR expression within the PL in the female rats on WD1. Unfortunately, we did not assay for estrous cycle stage during our neuropharmacological study to avoid confounding our examination of NBQX carry-over effects. Nevertheless, our neuropharmacological studies using the 1.0 𝜇g/side NBQX dose argue that AMPAR/KARs within mPFC undergo some form of plasticity over the course of cocaine withdrawal that changes the functional import of receptor activation for cue-elicited cocaine-seeking behavior. While not yet assayed within a model of incubated cocaine-craving, a time-dependent insertion of CP-AMPARs are reported to occur within layer 5 of mPFC pyramidal neurons of cocaine-sensitized mice during withdrawal, which impairs normal mGlu1/mTOR-dependent long-term depression in this region and induces a “malplastic state” (40). Indeed, prior neuropharmacological studies from our group have implicated both PI3K/Akt/mTOR activation (11, 47) and reduced mGlu1 function within mPFC (43) as critical molecular adaptations, respectively, for the expression and persistence of incubated cocaine-craving.

Now that we have confirmed an attenuating effect of intra-PL NBQX on incubated cocaine-seeking, future studies will seek to determine the relative contribution of these different iGluRs by comparing the effects of selective KAR versus AMPAR antagonists on cue-elicited cocaine-craving in early versus later withdrawal. Pending outcomes of these studies, the specific contribution of CP-AMPARs within the PL to incubated craving can be determined using the selective GluA2-lacking AMPAR antagonist Naspm. As inhibition of glutamate release within the IL also dampens incubated cocaine-craving (22), it will be important also to assay the relative role for different iGluRs expressed within the IL as driving cocaine-craving in the short and longer term. Finally, the inhibitory effect of intra-NAc Naspm extends from models of incubated cocaine-craving (e.g., 34, 38, 39), to those of methamphetamine- and oxycodone-craving (50, 68, 69), raising the possibility that withdrawal-dependent changes in specific AMPAR or KAR subtypes may also contribute to perturbations in synaptic plasticity within mPFC produced by other drugs of abuse.

### No overt changes in GluA1 and GluA2 expression associated with incubated cocaine-craving

Prior immunoblotting studies by our group indicated that the detection and magnitude of incubation-related changes in the expression of certain proteins within mPFC (e.g., Akt activation, mGlu1, mGlu5 and Homer2) are more robust in rats with a prior history of extended- versus shorter-access to intravenous cocaine (e.g., 8, 47 vs. 11, 43, 73). Indeed, evidence also indicates that the duration of daily cocaine-access impacts the ability to detect CP-AMPAR-related changes, at least within the NAc (Purgianto et al., 2013). However, the null results for GluA1 and GluA2 expression within the mPFC of the cocaine-incubated rats in the present study (**Figure 3**) align with those reported previously for rats expressing incubated cocaine-craving following shorter-access self-administration paradigms (8), arguing against the duration of cocaine-access as being a critical factor in our inability to detect changes in AMPAR subunit expression.

Prior studies of AMPAR subunit expression within the NAc have reported increased cell surface *and* intracellular GluA1 expression in cocaine-incubated rats (38, 74, 75) that is purported to reflect up-regulated GluA1 translation (e.g., 76–78). Based on the results of Conrad et al. (38), we rationalized that if incubated cocaine-craving was associated with increased surface and intracellular GluA1 expression, then our conventional immunoblotting procedures, conducted on whole-cell lysates, would be sufficient to detect changes in subunit expression within mPFC if they occurred. Indeed, we successfully detected a time-dependent increase in GluA1 expression within the IL of male rats exhibiting incubated sucrose-seeking (**Figure 5C**). Thus, it may be that (1) incubated cocaine-craving is completely dissociated from changes in AMPAR expression within mPFC or (2) the changes in AMPAR expression associated with the cocaine-incubated state are too subtle to detect in whole-cell lysates. Given on our null results (8; present study), we propose that any future immunoblotting studies of AMPAR subunit expression within mPFC employ subcellular fractionation or biotinylation procedures to isolate cell surface subunit expression to better inform the relationship between incubated cocaine-craving and the subunit composition of functionally relevant AMPARs, particularly in light of electrophysiological evidence supporting the insertion of GluA2-lacking AMPARs within mPFC pyramidal neurons during cocaine withdrawal (40).

### Estrous phase influences cue-induced cocaine-craving but not AMPAR expression

The magnitude of incubated cocaine-craving by female rats can be influenced by their hormonal status with heightened craving observed in the estrus phase compared to both females in non-estrus phases and males. Consistent with these prior studies, the female rats identified in the present study as being in estrus were the only females that exhibited incubated cocaine-craving, but these same estrus females were the only females exhibiting a time-dependent increase in inactive lever-responding (**Figures 4A, B**), suggestive of an estrus-related increase in overall behavioral reactivity to re-exposure to the cocaine-taking context following protracted withdrawal. How the estrous cycle impacts the biomolecular correlates within the mPFC of incubated cocaine-craving is not known. As such, we examined how GluA1 and GluA2 expression might vary with cycle phase in cocaine-experienced females. We failed to detect differences in total subunit expression as a function of the estrous cycle phase. However, we did detect a significant time-dependent increase in the relative expression of GluA2 versus GluA1 within the IL of the cocaine-experienced females in diestrus, while the relative expression of these subunits tended to decline with the passage of time in estrus or proestrus females (**Figure 4H**). Although cursory given that we did not power our study to detect estrous cycle phase effects, these data suggest a lower and higher prevalence of CP-AMPARs within the IL, respectively, during diestrus versus estrus/proestrus, at least in cocaine-incubated rats (**Figures 4A, B**). When examined within the IL of cocaine-naive females, non-NMDA excitatory postsynaptic currents are elevated in diestrus females, relative to both proestrus females and male controls and these currents were not inwardly rectifying, suggesting that diestrus is associated with increased CP-AMPAR expression within the IL of cocaine-naive females (79). Given that our cursory findings seem at odds with the results of this prior study, a more concerted effort is clearly required to understand how estrous cycle phase influences not only iGluR subunit expression, but also their cell surface expression and channel conductance, within mPFC subregions of cocaine-naive females versus cocaine-experienced rats tested for craving in early and protracted withdrawal, particularly in light of clear evidence that the magnitude of incubated cocaine-craving by females depends upon estrous cycle phase (**Figure 4A**; 48, 49, 52).

### Sex-selective protein correlates of incubated sucrose-seeking

The similar temporal profile of incubated craving for different drugs of abuse and non-drug reinforcers (e.g., sucrose, saccharin and high-fat foods) have led to the theory that common neurocircuitry and biomolecular changes might underpin the phenomenon of incubated craving (c.f., 80). Indeed, several studies have examined for common biomolecular mechanisms in the incubation of craving for drug versus non-drug reinforcers (81–83). Of relevance to the present study, increased indices of neuronal activity are reported within the PL and IL of cocaine-, heroin-, and sucrose-experienced rats (9, 84, 85), suggesting that these mPFC subregions are components of a common neurocircuitry driving incubated cue-elicited reward-seeking. However, a recent study by our group failed to identify “cocaine-like” changes in several glutamate-related proteins within mPFC subregions of rats expressing incubated sucrose-craving (41 vs. 10, 11, 43, 47, 73). Aligning with discrepancies in protein expression, we detected no changes in the total protein expression of either AMPAR subunit within the PL or IL in cocaine-incubated rats (**Figure 3**) and the only notable outcome associated with incubated cocaine-craving was a time-dependent increase in the GluA2:GluA1 ratio detected in diestrus females (**Figure 4H**) discussed above. In contrast, incubated sucrose-craving was associated with a male-selective increase in GluA1 expression within the IL (**Figure 5D**) and a female-selective increase in the relative expression of GluA2 within the PL (**Figure 5C**).

Our data to date indicate that the capacity of sucrose-associated cues to elevate extracellular glutamate levels within the mPFC dissipates, rather than intensifies, in male rats with the passage of time in withdrawal (21). As the microdialysis probes were situated at the interface of the PL and IL in our earlier study and this study employed male subjects only (21), the subregional and sex specificity of sucrose cue-elicited glutamate release is not known. However, our present results suggest that these changes may be subregionally selective, as well as sex-dependent. It is worth noting that the male-selectivity of the observed change in IL GluA1 expression in sucrose-incubated males aligns with our recent finding that incubated sucrose-craving *in these same males* is associated with increased IL expression of p(Ser473)-Akt, p(Ser729)-PKCε, and p(Ser2448)-mTOR (41), arguing that the present result for IL GluA1 expression is not likely spurious. In contrast to males, the same female rats as those employed in the present study exhibit lower total protein expression of p(Ser473)-Akt and p(Ser729)-PKCε within the PL (41), concomitant with elevated relative GluA2 expression (**Figure 5C**). Thus, despite the fact that the magnitude of incubated sucrose-craving is comparable between male and female rats (**Figure 5, top**; 41), our immunoblotting data to date indicate that the biomolecular mechanisms within mPFC driving incubated sucrose-craving are sex-dependent, both with respect to the specific signaling molecules affected and the subregion in which these changes occur. As an increase in GluA1 subunit expression is associated with the insertion of CP-AMPARs (e.g., 38, 74), the present results from males argue that incubated sucrose-craving may involve increased CP-AMPAR-mediated signaling within the IL specifically. Conversely, higher relative GluA2 expression is indicative of more CI-AMPARs (e.g., 38), arguing that incubated sucrose-craving in females may be driven moreso by CI-AMPARs within the PL. Thus, an important goal for future work is to determine whether sex differences exist in the effects of the GluA2-lacking AMPAR antagonist Naspm on the expression of incubated sucrose-craving.

## Conclusions

AMPAR/KAR inhibition within the PL blocked incubated cocaine-craving during protracted withdrawal in rats of both sexes, while increasing cocaine cue-reactivity in female rats during early withdrawal. Although AMPAR/KAR activation within the PL is clearly necessary for the expression of incubated cocaine-craving, incubated cocaine-craving is not overtly related to the total or relative expression of GluA1 and GluA2 subunits within either the PL or IL. In contrast, increased GluA1 expression within the IL and increased relative expression of GluA2 within the PL is associated with incubated sucrose-craving, respectively in male and female rats. These data indicate a key role for PL AMPAR/KARs in driving incubated cocaine-craving and suggest that AMPARs may potentially gate the development of incubated sucrose-craving in a sex- and subregion-selective manner. Our findings inform as to the biomolecular mechanisms within mPFC that drive incubated craving across drug and non-drug reinforcers of relevance to both the efficacy and side-effect profiles of glutamate-targeting therapies for treating pathological craving in both cocaine use and eating disorders.

## Data Availability

The raw data supporting the conclusions of this article will be made available by the authors, without undue reservation.

## References

[1] Substance Abuse and Mental Health Services Administration. 2023 Companion infographic report: Results from the 2021, 2022, and 2023 National Surveys on Drug Use and Health (SAMHSA Publication No. PEP24-07-020). In: Center for behavioral health statistics and quality, substance abuse and mental health services (2024). Available online at: https://www.samhsa.gov/data/report/2021-2022-2023-nsduh-infographic.

[2] Grimm JW Hope BT Wise RA Shaham Y. Incubation of cocaine craving after withdrawal. Nature. (2001) 412:141–2. doi: 10.1038/35084134, PMID: 11449260 PMC2889613

[3] Chow JJ Pitts KM Negishi K Madangopal R Dong Y Wolf ME. Neurobiology of the incubation of drug craving: An update. Pharmacol Rev. (2025) 77:100022. doi: 10.1016/j.pharmr.2024.100022, PMID: 40148031 PMC11964951

[4] Dong Y Taylor JR Wolf ME Shaham Y. Circuit and synaptic plasticity mechanisms of drug relapse. J Neurosci. (2017) 37:10867–76. doi: 10.1523/JNEUROSCI.1821-17.2017, PMID: 29118216 PMC5678019

[5] Devoto F Zapparoli L Spinelli G Scotti G Paulesu E. How the harm of drugs and their availability affect brain reactions to drug cues: a meta-analysis of 64 neuroimaging activation studies. Transl Psychiatry. (2020) 10:429. doi: 10.1038/s41398-020-01115-7, PMID: 33318467 PMC7736294

[6] Goldstein RZ Volkow ND. Dysfunction of the prefrontal cortex in addiction: neuroimaging findings and clinical implications. Nat Rev Neurosci. (2011) 12:652–69. doi: 10.1038/nrn3119, PMID: 22011681 PMC3462342

[7] Mohd Nawawi NAA Nasir F Abdullah KA Othman E. Understanding drug craving: evidence from fMRI studies. Neurosci Res Notes 7(3). (2024) 294:1–294.10. doi: 10.31117/neuroscirn.v7i3.294

[8] Huerta Sanchez LL Sankaran M Li TL Doan H Chiu A Shulman E. Profiling prefrontal cortex protein expression in rats exhibiting an incubation of cocaine craving following short-access self-administration procedures. Front Psychiatry 13. (2023) 1031585:1031585. doi: 10.3389/fpsyt.2022.1031585, PMID: 36684008 PMC9846226

[9] Koya E Uejima JL Wihbey KA Bossert JM Hope BT Shaham Y. Role of ventral medial prefrontal cortex in incubation of cocaine craving. Neuropharmacology. 56. (2009) 56(Suppl 1):177–85. doi: 10.1016/j.neuropharm.2008.04.022, PMID: 18565549 PMC2635336

[10] Miller BW Wroten MG Sacramento AD Silva HE Shin CB Vieira PA. Cocaine craving during protracted withdrawal requires pkcϵ priming within. Addiction biology (2016) 22(3):629–39. doi: 10.1111/adb.12354, PMID: 26769453 PMC4945488

[11] Szumlinski KK Ary AW Shin CB Wroten MG Courson J Miller BW. PI3K activation within ventromedial prefrontal cortex regulates the expression of drug-seeking in two rodent species. Addict Biol. (2019) 24:1216–26. doi: 10.1111/adb.12696, PMID: 30450839 PMC6849400

[12] Kalivas PW Volkow N Seamans J. Unmanageable motivation in addiction: A pathology in prefrontal-accumbens glutamate transmission. Neuron. (2005) 45:647–50. doi: 10.1016/j.neuron.2005.02.005, PMID: 15748840

[13] Manoocheri Carter AG. Rostral and caudal basolateral amygdala engage distinct circuits in the prelimbic and infralimbic prefrontal cortex. eLife. (2022) 11:e82688. doi: 10.7554/eLife.82688, PMID: 36476757 PMC9803354

[14] Sesack SR Deutch AY Roth RH Bunney BS. Topographical organization of the efferent projections of the medial prefrontal cortex in the rat: an anterograde tract-tracing study with Phaseolus vulgaris leucoagglutinin. J Comp Neurol. (1989) 290:213–42. doi: 10.1002/cne.902900205, PMID: 2592611

[15] Sun XY Liu L Song YT Wu T Zheng T Hao JR. Two parallel medial prefrontal cortex-amygdala pathways mediate memory deficits via glutamatergic projection in surgery mice. Cell Rep. (2023) 42:112719. doi: 10.1016/j.celrep.2023.112719, PMID: 37392387

[16] Gabbott PL Dickie BG Vaid RR Headlam AJ Bacon SJ. Local-circuit neurones in the medial prefrontal cortex (areas 25, 32 and 24b) in the rat: morphology and quantitative distribution. J Comp Neurol. (1997) 377:465–99. doi: 10.1002/(sici)1096-9861(19970127)377:4<465::aid-cne1>3.0.co;2-0, PMID: 9007187

[17] Geisler S Derst C Veh RW Zahm DS. Glutamatergic afferents of the ventral tegmental area in the rat. J Neurosci. (2007) 27:5730–43. doi: 10.1523/JNEUROSCI.0012-07.2007, PMID: 17522317 PMC3202987

[18] Jay TM Glowinski J Thierry AM. Selectivity of the hippocampal projection to the prelimbic area of the prefrontal cortex in the rat. Brain Res. (1989) 505:337–40. doi: 10.1016/0006-8993(89)91464-9, PMID: 2598054

[19] Jefferson T Kelly CJ Martina M. Differential rearrangement of excitatory inputs to the medial prefrontal cortex in chronic pain models. Front Neural Circuits. (2021) 15:791043. doi: 10.3389/fncir.2021.791043, PMID: 35002635 PMC8738091

[20] Sun T Song Z Tian Y Tian W Zhu C Ji G. Basolateral amygdala input to the medial prefrontal cortex controls obsessive-compulsive disorder-like checking behavior. Proc Natl Acad Sci U.S.A. (2019) 116:3799–804. doi: 10.1073/pnas.1814292116, PMID: 30808765 PMC6397577

[21] Shin CB Serchia MM Shahin JR Ruppert-Majer MA Kippin TE Szumlinski KK. Incubation of cocaine-craving relates to glutamate over-flow within ventromedial prefrontal cortex. Neuropharmacology. (2016) 102:103–10. doi: 10.1016/j.neuropharm.2015.10.038, PMID: 26522436 PMC4698200

[22] Shin CB Templeton TJ Chiu AS Kim J Gable ES Vieira PA. Endogenous glutamate within the prelimbic and infralimbic cortices regulates the incubation of cocaine-seeking in rats. Neuropharmacology. (2018) 128:293–300. doi: 10.1016/j.neuropharm.2017.10.024, PMID: 29061508 PMC6400061

[23] Kalivas PW. The glutamate homeostasis hypothesis of addiction. . Nat Rev Neurosci. (2009) 10:561–72. doi: 10.1038/nrn2515, PMID: 19571793

[24] Kalivas PW McFarland K. Brain circuitry and the reinstatement of cocaine-seeking behavior. Psychopharmacology. (2003) 168:44–56. doi: 10.1007/s00213-003-1393-2, PMID: 12652346

[25] LaLumiere RT Smith KC Kalivas PW. Neural circuit competition in cocaine-seeking: roles of the infralimbic cortex and nucleus accumbens shell. Eur J Neurosci. (2012) 35:614–22. doi: 10.1111/j.1460-9568.2012.07991.x, PMID: 22321070 PMC3281521

[26] Peters J LaLumiere RT Kalivas PW. Infralimbic prefrontal cortex is responsible for inhibiting cocaine seeking in extinguished rats. J neuroscience: Off J Soc Neurosci. (2008) 28:6046–53. doi: 10.1523/JNEUROSCI.1045-08.2008, PMID: 18524910 PMC2585361

[27] Ma YY Lee BR Wang X Guo C Liu L Cui R. Bidirectional modulation of incubation of cocaine craving by silent synapse-based remodeling of prefrontal cortex to accumbens projections. Neuron. (2014) 83:1453–67. doi: 10.1016/j.neuron.2014.08.023, PMID: 25199705 PMC4295617

[28] Diering GH Huganir RL. The AMPA receptor code of synaptic plasticity. Neuron. (2018) 100:314–29. doi: 10.1016/j.neuron.2018.10.018, PMID: 30359599 PMC6214363

[29] Toda S McGinty JF Kalivas PW. Repeated cocaine administration alters the expression of genes in corticolimbic circuitry after a 3-week withdrawal: a DNA macroarray study. J Neurochem. (2002) 82:1290–9. doi: 10.1046/j.1471-4159.2002.01083.x, PMID: 12358776

[30] Hemby SE Horman B Tang W. Differential regulation of ionotropic glutamate receptor subunits following cocaine self-administration. Brain Res. (2005) 1064:75–82. doi: 10.1016/j.brainres.2005.09.051, PMID: 16277980 PMC3843347

[31] Tang W Wesley M Freeman WM Liang B Hemby SE. Alterations in ionotropic glutamate receptor subunits during binge cocaine self-administration and withdrawal in rats. J Neurochem. (2004) 89:1021–33. doi: 10.1111/j.1471-4159.2004.02392.x, PMID: 15140200 PMC3843358

[32] Tang WX Fasulo WH Mash DC Hemby SE. Molecular profiling of midbrain dopamine regions in cocaine overdose victims. J Neurochem. (2003) 85:911–24. doi: 10.1046/j.1471-4159.2003.01740.x, PMID: 12716423 PMC3843357

[33] Hemby SE Tang W Muly EC Kuhar MJ Howell L Mash DC. Cocaine-induced alterations in nucleus accumbens ionotropic glutamate receptor subunits in human and non-human primates. J Neurochem. (2005) 95:1785–93. doi: 10.1111/j.1471-4159.2005.03517.x, PMID: 16363995 PMC3843355

[34] Loweth JA Tseng KY Wolf ME. Adaptations in AMPA receptor transmission in the nucleus accumbens contributing to incubation of cocaine craving. Neuropharmacology 76 Pt B(0. (2014) 0):287–300. doi: 10.1016/j.neuropharm.2013.04.061, PMID: 23727437 PMC3836860

[35] Di Ciano P Cardinal RN Cowell RA Little SJ Everitt BJ. Differential involvement of NMDA, AMPA/kainate, and dopamine receptors in the nucleus accumbens core in the acquisition and performance of pavlovian approach behavior. J Neurosci. (2001) 21:9471–7. doi: 10.1523/JNEUROSCI.21-23-09471.2001, PMID: 11717381 PMC6763894

[36] Doyle SE Ramôa C Garber G Newman J Toor Z Lynch WJ. A shift in the role of glutamatergic signaling in the nucleus accumbens core with the development of an addicted phenotype. Biol Psychiatry. (2014) 76:810–5. doi: 10.1016/j.biopsych.2014.02.005, PMID: 24629536 PMC4133320

[37] Lynch WJ Bakhti-Suroosh A Abel JM Davis C. Shifts in the neurobiological mechanisms motivating cocaine use with the development of an addiction-like phenotype in male rats. Psychopharmacology. (2021) 238:811–23. doi: 10.1007/s00213-020-05732-4, PMID: 33241478 PMC8290931

[38] Conrad KL Tseng KY Uejima JL Reimers JM Heng LJ Shaham Y. Formation of accumbens GluR2-lacking AMPA receptors mediates incubation of cocaine craving. Nature. (2008) 454:118–21. doi: 10.1038/nature06995, PMID: 18500330 PMC2574981

[39] Kawa AB Hwang EK Funke JR Zhou H Costa-Mattioli M Wolf ME. Positive allosteric modulation of mGlu1 reverses cocaine-induced behavioral and synaptic plasticity through the integrated stress response and oligophrenin-1. Biol Psychiatry. (2022) 92:871–9. doi: 10.1016/j.biopsych.2022.05.008, PMID: 35871097 PMC10656746

[40] Ruan H Yao WD. Loss of mGluR1-LTD following cocaine exposure accumulates Ca2+-permeable AMPA receptors and facilitates synaptic potentiation in the prefrontal cortex. J Neurogenetics. (2021) 35:358–69. doi: 10.1080/01677063.2021.1931180, PMID: 34092163 PMC9255266

[41] Cano FJ Denning CJE Udayashankar H Adler SD Smith KE Dang V. Biomolecular Correlates of Incubated Sucrose-Seeking within Ventromedial Prefrontal Cortex are Sex- and Subregion-Selective. (2025). doi: 10.2139/ssrn.5239606. PMC12884955 40850562

[42] Purgianto A Scheyer AF Loweth JA Ford KA Tseng KY Wolf ME. Different adaptations in AMPA receptor transmission in the nucleus accumbens after short vs long access cocaine self-administration regimens. Neuropsychopharmacology. (2013) 38(9):1789–97. doi: 10.1038/npp.2013.78, PMID: 23546386 PMC3717554

[43] Ben-Shahar O Sacramento AD Miller BW Webb SM Wroten MG Silva HE. Deficits in ventromedial prefrontal cortex group 1 metabotropic glutamate receptor function mediate resistance to extinction during protracted withdrawal from an extensive history of cocaine self-administration. J Neurosci. (2013) 33:495–506a. doi: 10.1523/JNEUROSCI.3710-12.2013, PMID: 23303930 PMC3711633

[44] Randle JC Guet T Cordi A Lepagnol JM. Competitive inhibition by NBQX of kainate/AMPA receptor currents and excitatory synaptic potentials: importance of 6-nitro substitution. Eur J Pharmacol. (1992) 215:237–44. doi: 10.1016/0014-2999(92)90033-z, PMID: 1382998

[45] Russell SE Puttick DJ Sawyer AM Potter DN Mague S Carlezon W. A. C.OMMAJ.R.X.X.X. Nucleus accumbens AMPA receptors are necessary for morphine-withdrawal-induced negative-affective states in rats. J Neurosci. (2016) 36:5748–62. doi: 10.1523/JNEUROSCI.2875-12.2016, PMID: 27225765 PMC4879196

[46] Biondo AM Clements RL Hayes DJ Eshpeter B Greenshaw AJ. NMDA or AMPA/kainate receptor blockade prevents acquisition of conditioned place preference induced by D(2/3) dopamine receptor stimulation in rats. Psychopharmacology. (2005) 179:189–97. doi: 10.1007/s00213-005-2201-y, PMID: 15744543

[47] Chiu AS Kang MC Huerta Sanchez LL Fabella AM Holder KN Barger BD. Preclinical evidence to support repurposing everolimus for craving reduction during protracted drug withdrawal. Neuropsychopharmacology : official publication of the American College of Neuropsychopharmacology. (2021) 46:2090–100. doi: 10.1038/s41386-021-01064-9, PMID: 34188183 PMC8505628

[48] Corbett CM Dunn E Loweth JA. Effects of sex and estrous cycle on the time course of incubation of cue-induced craving following extended-access cocaine self-administration. . eNeuro. (2021) 8:ENEURO.0054–21.2021. doi: 10.1523/ENEURO.0054-21.2021, PMID: 34290059 PMC8362687

[49] Kerstetter KA Su Z-I Ettenberg A Kippin TE. Protracted time-dependent increases in cocaine-seeking behavior during cocaine withdrawal in female relative to male rats. Psychopharmacology. (2013) 198:63–75. doi: 10.1007/s00213-008-1089-8, PMID: 18265959

[50] Nicholas AP Loweth JA Tseng KY. AMPA receptor and metabotropic glutamate receptor 1 adaptations in the nucleus accumbens core during incubation of methamphetamine craving. Neuropsychopharmacology. (2019) 44:1534–41. doi: 10.1038/s41386-019-0425-5, PMID: 31146278 PMC6785134

[51] Tada H Koide M Ara W Shibata Y Funabashi T Suyama K. Estrous cycle-dependent phasic changes in the stoichiometry of hippocampal synaptic AMPA receptors in rats. PloS One. (2015) 10:e0131359. doi: 10.1371/journal.pone.0131359, PMID: 26121335 PMC4486186

[52] Nicolas C Russell TI Pierce AF Maldera S Holley A You ZB. Incubation of cocaine craving after intermittent-access self-administration: sex differences and estrous cycle. Biol Psychiatry. (2019) 85:915–24. doi: 10.1016/j.biopsych.2019.01.015, PMID: 30846301 PMC6534474

[53] Rozov A Burnashev N. Polyamine-dependent facilitation of postsynaptic AMPA receptors counteracts paired-pulse depression. Nature. (1999) 401:594–8. doi: 10.1038/44151, PMID: 10524627

[54] Wolf ME. Targeting neuroplasticity in substance use disorders: implications for therapeutics. Annu Rev Pharmacol Toxicol. (2025) 65:259–280. 82. doi: 10.1146/annurev-pharmtox-061724-080548, PMID: 39374445 PMC11864087

[55] Bowers MS Chen BT Bonci A. AMPA receptor synaptic plasticity induced by psychostimulants: the past, present, and therapeutic future. Neuron. (2010) 67:11–24. doi: 10.1016/j.neuron.2010.06.004, PMID: 20624588 PMC2904302

[56] Gregus AM Tropea TF Wang Y Hauck SC Costa AC Rajadhyaksha AM. Deletion of the GluR5 subunit of kainate receptors affects cocaine sensitivity and preference. Neurosci Lett. (2010) 468:86–9. doi: 10.1016/j.neulet.2009.10.071, PMID: 19878705 PMC2815225

[57] Cornish JL Kalivas PW. Glutamate transmission in the nucleus accumbens mediates relapse in cocaine addiction. J Neurosci. (2000) 20:RC89. doi: 10.1523/JNEUROSCI.20-15-j0006.2000, PMID: 10899176 PMC6772531

[58] Menniti FS Buchan AM Chenard BL Critchett DJ Ganong AH Guanowsky V. CP-465,022, a selective noncompetitive AMPA receptor antagonist, blocks AMPA receptors but is not neuroprotective in *vivo* . Stroke. (2003) 34:171–6. doi: 10.1161/01.str.0000048216.90221.9c, PMID: 12511770

[59] Rogawski MA Hanada T. Preclinical pharmacology of perampanel, a selective non-competitive AMPA receptor antagonist. Acta Neurol Scand Suppl. (2013) 197:19–24. doi: 10.1111/ane.12100, PMID: 23480152 PMC4506647

[60] Dargan SL Clarke VR Alushin GM Sherwood JL Nisticò R Bortolotto ZA. ACET is a highly potent and specific kainate receptor antagonist: characterisation and effects on hippocampal mossy fibre function. Neuropharmacology. (2009) 56:121–30. doi: 10.1016/j.neuropharm.2008.08.016, PMID: 18789344 PMC2637447

[61] More JC Nistico R Dolman NP Clarke VR Alt AJ Ogden AM. Characterisation of UBP296: a novel, potent and selective kainate receptor antagonist. Neuropharmacology. (2004) 47:46–64. doi: 10.1016/j.neuropharm.2004.03.005, PMID: 15165833

[62] Akiyama K Ujike H Sakai K Shimizu Y Kodama M Kuroda S. Effect of 2,3-dihydroxy-6-nitro-7-sulfamoyl-benzo(f)quinoxaline on methamphetamine- and cocaine-induced behavioral sensitization. Pharmacol Biochem Behav. (1998) 61(4):419–26. doi: 10.1016/s0091-3057(98)00121-x, PMID: 9802837

[63] Bäckström P Hyytiä P. Ionotropic and metabotropic glutamate receptor antagonism attenuates cue-induced cocaine seeking. Neuropsychopharmacology. (2006) 31:778–86. doi: 10.1038/sj.npp, PMID: 16123768

[64] Boldry RC Kelland MD Engber TM Chase TN. NBQX inhibits AMPA-induced locomotion after injection into the nucleus accumbens. Brain Res. (1993) 600:331–4. doi: 10.1016/0006-8993(93)91392-6, PMID: 7679606

[65] Faccidomo S Eastman VR Santanam TS Swaim KS Taylor SM Hodge CW. Distinct sex differences in ethanol consumption and operant self-administration in C57BL/6J mice with uniform regulation by glutamate AMPAR activity. Front Behav Neurosci. (2025) 18:1498201. doi: 10.3389/fnbeh.2024.1498201, PMID: 39911242 PMC11794300

[66] Stephens DN Brown G. Disruption of operant oral self-administration of ethanol, sucrose, and saccharin by the AMPA/kainate antagonist, NBQX, but not the AMPA antagonist, GYKI 52466. Alcohol Clin Exp Res. (1999) 23:1914–20. doi: 10.1097/00000374-199912000-00009, PMID: 10630610

[67] Vanover KE. Effects of AMPA receptor antagonists on dopamine-mediated behaviors in mice. Psychopharmacol (Berl). (1998) 136:123–31. doi: 10.1007/s002130050547, PMID: 9551768

[68] Scheyer AF Loweth JA Christian DT Uejima J Rabei R Le T. AMPA receptor plasticity in accumbens core contributes to incubation of methamphetamine craving. Biol Psychiatry. (2016) 80:661–70. doi: 10.1016/j.biopsych.2016.04.003, PMID: 27264310 PMC5050076

[69] Wong B Zimbelman AR Milovanovic M Wolf ME Stefanik MT. GluA2-lacking AMPA receptors in the nucleus accumbens core and shell contribute to the incubation of oxycodone craving in male rats. Addict Biol. (2022) 27:e13237. doi: 10.1111/adb.13237, PMID: 36301206 PMC10655598

[70] Hoffman JL Faccidomo S Saunders BL Taylor SM Kim M Hodge CW. (2021)Inhibition of AMPA receptors (AMPARs) containing transmembrane AMPAR regulatory protein γ-8 with JNJ-55511118 shows preclinical efficacy in reducing chronic repetitive alcohol self-administration. Alcohol Clin Exp Res. 45:1424–35. doi: 10.1111/acer.14639, PMID: 34086361 PMC8336716

[71] Ferrario CR Loweth JA Milovanovic M Ford KA Galiñanes GL Heng LJ. Alterations in AMPA receptor subunits and TARPs in the rat nucleus accumbens related to the formation of Ca²^+^-permeable AMPA receptors during the incubation of cocaine craving. Neuropharmacology. (2011) 61:1141–51. doi: 10.1016/j.neuropharm.2011.01.021, PMID: 21276808 PMC3094740

[72] Palomero-Gallagher N Bidmon HJ Zilles K. AMPA, kainate, and NMDA receptor densities in the hippocampus of untreated male rats and females in estrus and diestrus. J Comp Neurol. (2003) 459:468–74. doi: 10.1002/cne.10638, PMID: 12687711

[73] Gould AT Sacramento AD Wroten MG Miller BW von Jonquieres G Klugmann M. Cocaine-elicited imbalances in ventromedial prefrontal cortex Homer1 versus Homer2 expression: implications for relapse. Addict Biol. (2015) 20:148–57. doi: 10.1111/adb.12088, PMID: 24118426 PMC3969898

[74] Boudreau AC Reimers JM Milovanovic M Wolf ME. Cell surface AMPA receptors in the rat nucleus accumbens increase during cocaine withdrawal but internalize after cocaine challenge in association with altered activation of mitogen-activated protein kinases. J Neurosci. (2007) 27:10621–35. doi: 10.1523/JNEUROSCI.2163-07.2007, PMID: 17898233 PMC2856315

[75] Reimers JM Milovanovic M Wolf ME. Quantitative analysis of AMPA receptor subunit composition in addiction-related brain regions. Brain Research. (2011) 1367:223–33. doi: 10.1016/j.brainres.2010.10.016, PMID: 20946890 PMC3005033

[76] Scheyer AF Wolf ME Tseng KY. A protein synthesis-dependent mechanism sustains calcium-permeable AMPA receptor transmission in nucleus accumbens synapses during withdrawal from cocaine self-administration. J Neurosci. (2014) 34(8):3095–100. doi: 10.1523/JNEUROSCI.4940-13.2014, PMID: 24553949 PMC3929765

[77] Hwang EK Wunsch AM Wolf ME. Retinoic acid-mediated homeostatic plasticity drives cell type-specific CP-AMPAR accumulation in nucleus accumbens core and incubation of cocaine craving. Mol Psychiatry. (2025) 30(7):3175–87. doi: 10.1038/s41380-025-03026-9, PMID: 40316677 PMC12228501

[78] Stefanik MT Milovanovic M Werner CT Spainhour JCG Wolf ME. Withdrawal from cocaine self-administration alters the regulation of protein translation in the nucleus accumbens. Biol Psychiatry. (2018) 84:223–32. doi: 10.1016/j.biopsych.2018.02.012, PMID: 29622268 PMC6054574

[79] Galvin C Ninan I. Regulation of the mouse medial prefrontal cortical synapses by endogenous estradiol. Neuropsychopharmacology. (2014) 39:2086–94. doi: 10.1038/npp.2014.56, PMID: 24608267 PMC4104325

[80] Grimm JW. Incubation of food craving in rats: A review. J Expt Anal Behav. (2020) 113:37–47. doi: 10.1002/jeab.561, PMID: 31709556 PMC8034663

[81] Knackstedt LA Kalivas PW. Time-dependent decreases in nucleus accumbens AMPA/NMDA ratio and incubation of sucrose craving in adolescent and adult rats. Psychopharmacology. (2014) 231:1681–9. doi: 10.1007/s00213-014-3582-4, PMID: 24114427 PMC3967069

[82] Blanco-Gandía MC Rodríguez-Arias M Miñarro J. Calcium-permeable AMPA receptors in the nucleus accumbens core mediate the incubation of cocaine craving. Psychopharmacology. (2020) 237:1457–69. doi: 10.1007/s00213-020-05579-0

[83] Venniro M Zhang M Shaham Y Caprioli D. Social choice-induced voluntary abstinence prevents incubation of cocaine craving. Addict Biol. (2021) 26:e12968. doi: 10.1111/adb.12968, PMID: 32985064 PMC8855307

[84] Counotte DS Schiefer C Shaham Y O’Donnell P. Time-dependent decreases in nucleus accumbens AMPA/NMDA ratio and incubation of sucrose craving in adolescent and adult rats. Psychopharmacology. (2013) 231:1675–84. doi: 10.1007/s00213-013-3294-3, PMID: 24114427 PMC3967069

[85] Grimm JW Hope BT Wise RA. Epac signaling is required for cocaine-induced change in AMPA receptor subunit composition in the ventral tegmental area. J Neurosci. (2016) 36:12345–57. doi: 10.1523/JNEUROSCI.3024-16.2016, PMID: 27122037 PMC4846675

